# AKAP8L enhances the stemness and chemoresistance of gastric cancer cells by stabilizing SCD1 mRNA

**DOI:** 10.1038/s41419-022-05502-4

**Published:** 2022-12-15

**Authors:** Ruihong Zhang, Luguang Liu, Fengqin Wang, Weizhu Zhao, Kai Liu, Hang Yu, Siwei Zhao, Botao Xu, Xiaoli Zhang, Jie Chai, Jing Hao

**Affiliations:** 1 Key Laboratory of The Ministry of Education for Experimental Teratology, Department of Histology and Embryology, School of Basic Medical Sciences, Cheeloo College of Medicine, Shandong University, 44 Wenhua Xi Road, Jinan, Shandong P. R. China; 2 Department of Breast and Thyroid Surgery, Shandong Provincial Hospital, Cheeloo College of Medicine, Shandong University, 324 Jingwuweiqi Road, Jinan, Shandong P. R. China; 3 Department of Gastrointestinal Surgery, Shandong Cancer Hospital and Institute, Shandong First Medical University and Shandong Academy of Medical Sciences, 440 Jiyan Road, Jinan, Shandong P. R. China; 4 Advanced Medical Research Institute, Cheeloo College of Medicine, Shandong University, 44 Wenhua Xi Road, Jinan, Shandong P. R. China; 5 Department of Oncology, Binzhou People’s Hospital, 515 Huangheqi Road, Binzhou, Shandong P. R. China

**Keywords:** Cell growth, Cancer stem cells

## Abstract

Gastric cancer (GC) remains the third leading cause of cancer-related deaths. Chemoresistance is the major determinant of GC treatment failure. To explore the molecular mechanisms of GC chemoresistance, mass spectrometry was performed to detect the genes altered in expression between chemoresistant and chemosensitive GC. PRKA kinase anchor protein 8L (AKAP-8L) was identified as one of the top upregulated genes in chemoresistant GC tissues. Moreover, the higher AKAP-8L expression was associated with the lower survival rate in GC patients. Overexpression of AKAP-8L enhanced the GC cell stemness and chemoresistance of oxaliplatin in vivo and in vitro. AKAP-8L deficiency obtained the opposite results. Mechanistically, AKAP-8L interacted with Stearoyl-CoA desaturase 1 (SCD1) mRNA and IGF2BP1 protein, and regulated SCD1 mRNA stability via IGF2BP1-dependent manner. SCD1 played a critical role in mediating the function of AKAP-8L in GC cell stemness and chemoresistance. Clinically, AKAP-8L and SCD1 protein levels was positively associated with human GC chemoresistance. Taken together, our results demonstrated that AKAP-8L facilitates GC chemoresistance via regulating SCD1-mediated stemness of GC cells. AKAP8L may represent a novel therapeutic target to overcome GC chemoresistance.

## Introduction

Gastric cancer remains one of the most common malignancies worldwide and the third leading cause of cancer-related deaths [1]. Because more than 60% of GC patients have developed local or distant metastasis at diagnosis, chemotherapy is the main choice for most of them [2–4]. Drug resistance is a major determinant leading to treatment failure and a low 5-year survival rate for patients with gastric cancer [5–7]. Oxaliplatin (Oxa) is commonly used in neoadjuvant chemotherapy to treat GC. Hence, understanding the key molecules involved in the complex process of tumor oxaliplatin chemoresistance is likely to contribute to the development of effective therapeutics for treating GC patients.

The A-kinase anchor proteins (AKAPs) are a group of structurally diverse proteins that have the common function of anchoring protein kinase A (PKA) to various cellular substrates by the interaction with the regulatory subunit [8, 9]. Because of the specificity of AKAPs in oncogenesis, they have been considered practical targets for cancer therapeutics. Besides localizing PKA, AKAP8L, a homologue of AKAP8, is also engaged in RNA processing and transcription [10]. AKAP8L interacts with mTORC1 to promote cell growth and cell proliferation [11]. However, the role of AKAP8L in GC chemoresistance and the underlying mechanism remains to be determined.

Here, we used unbiased approach to uncover novel modulators of chemoresistance by comparing gene expression between chemoresistant and chemosensitive gastric tumors. AKAP8L was demonstrated to play a critical role in GC stemness and chemoresistance in vitro as well as in vivo. Importantly, we found that AKAP8L interacts with SCD1 mRNA and modulates its mRNA stability via IGF2BP1- dependent manner in GC cells. Moreover, SCD1 appears to mediate the effects of AKAP8L on the stemness and chemoresistance of GC cells. Collectively, our study provided new insights into the potential of AKAP8L as a biomarker as well as a therapeutic target in the treatment of GC chemoresistance.

## Materials and methods

### Patient tissue samples

76 fresh gastric cancer specimens were collected from gastric cancer patients sensitive (31 cases) or resistant (45 cases) to chemotherapy from Shandong Cancer Hospital and Institute. The fresh tumor samples were preserved in liquid nitrogen immediately after resection. Each specimen was attached to a confirmed pathological diagnosis. All experiments in this study were endorsed by the Ethics Committee of School of Basic Medical Science of Shandong University and complied with the Declaration of Helsinki. Informed consent was obtained from all patients.

### Mass spectrometry

GC samples were measured by Intra-solution enzymatic lysis. Mass spectrometry analysis identified the differential proteins. HEK293T cells were transfected with Flag-AKAP8L, after 48 h, the anti-Flag antibody was added to enrich proteins binding to AKAP8L. Proteins were separated by SDS-polyacrylamide gel. Then, SDS-polyacrylamide gel was performed with Coomassie brilliant blue, and the binding proteins were measured by mass spectrometry (Maxis II, Advanced Medical Research Institute, Shandong University).

### Cell lines and culture

Human gastric cancer cell lines BGC-823 and MKN-45, and human embryonic kidney cell line-293 T, were obtained from the Cell Bank of the Chinese Academy of Sciences (Shanghai, China). All cells were maintained in DMEM supplemented with 10% FBS and actively passaged for less than 6 months from the time that they were received. The cell lines were authenticated by short tandem repeat (STR) profiling and tested free of mycoplasma.

### Construction of the Oxa-resistant BGC-823 and MKN-45 cells

BGC-823 and MKN-45 cells were seeded into the 6-well plates. After growing to 70~80% confluence, the media contained Oxa (10 mg/mL) was added into the wells. At 24 h after drug treatment, the drug-contained media was replaced by fresh media (without Oxa) after the cells were washed by phosphate-buffered saline (PBS) twice. The drug-contained media was again added at the second cell passage and the treatment time was prolonged gradually (for 48 and 72 h, respectively). The following process was the same as the aforementioned. Until the cell sensitivity to Oxa treatment was decreased obviously and became stable, MTT assays were performed to confirm the successful construction of the Oxa-resistant BGC-823 and MKN-45 cells (BGC-823/Oxa and MKN-45/Oxa).

### Cell transfection

Before transfection, the BGC-823/Oxa or MKN-45/Oxa cells were seeded in the 12-well plates. When growing to 30%–40% confluence, 1 mL fresh media supplemented with 10 μl polybrene (Obio technology) and 5 μl lentiviruses (LV-AKAP8L or LV-AKAP8L shRNA-1, shRNA-2) were added into each well. Puromycin (1 μg/mL) (Solarbio, P8230) was used to establish the stable AKAP8L overexpression or knockdown cell lines.

For knockdown of SCD1 or IGF2BP1, siRNAs against SCD1 or IGF2BP1 (siRNA), and a NC siRNA (Ribo, Guangzhou, China) were transfected into the BGC-823/Oxa cells using lipofectamine 2000 according to the manufacture’s protocol. After being incubated for 6~8 h, the cells were washed by PBS twice and the fresh media (supplemented with 10% FBS) were added for another 24–48 h incubation. Then the cells were collected and used for qPCR assay, Western blotting, MTT assay, sphere formation assay, and so on. The target sequences for shRNA or siRNA as follows: shAKAP8L-1: CCGCAGTATTCTCAACAACAA; shAKAP8L-2: CGTCACTAACAAGACCAAGAA; siSCD1: CTACGGCTCTTTCTGATCA; siIGF2BP1: GGCTCAGTATGGTACAGTA.

### MTT assay

In brief, 2 × 10^3^/mL suspended cells were plated per well in 96-well plates. After incubation for 24 h at 37 °C with 5% CO_2_, 100 μl drug-contained media were added into each well by certain concentration gradients for Oxa, 0, 5, 10, 15, 20, 25 μg/mL). 24 h later, add 10 μl MTT reagent (5 mg/mL) (Sigma) to per well and incubate for another 2~4 h. Then 150 μl/well dimethyl sulfoxides were used to terminate the reaction. After concussion for 10 min in a low speed, the absorbance values were recorded at 570 nm using an enzyme-linked immunometric meter (SpectraMax M5, USA).

### Mammosphere formation assay

BGC-823/Oxa or MKN-45/Oxa cells were plated in ultralow attachment six-well plates at a low density of 1 × 10^4^ viable cells/mL. Cells were maintained in DMEM supplemented with B27, 20 ng/mL EGF, and 20 ng/mL bFGF for two weeks. The mammospheres >50 μm were photographed and counted using inverted microscope.

### Soft agar colony formation assay

Soft agar assay was done by seeding cells at a density of 2 × 10^3^ in 24-well plate containing 0.3% top low-melt agarose and 0.6% bottom low-melt agarose. Cells were fed every 3 days, and colonies > 50 μm were counted using inverted microscope after two weeks.

### Flow cytometry analysis

To assess the cell apoptosis in vitro, cells were analyzed with Annexin V Apoptosis Detection kit (Vazyme Biotech,China) according to the manufacture’s instructions. Cells were examined by flow cytometry(CytoFLEX, Beckman Coulter).

### Animal study

Animal experiments were carried out according to the policy of the animal welfare and were approved by the Ethics Committee of School of Basic Medical Science of Shandong University. To construct the subcutaneous gastric tumor model, 1 × 10^6^ NC BGC-823/Oxa and AKAP8L-overexpression BGC-823/Oxa cells were, respectively, injected into 4 to 5-week-old nude mice after being diluted with PBS to 100 μl (the mice were bought from Weitong lihua, China). Five mice were used in each experimental group and nude mice were randomly divided into the control and experimental groups. The phenotype was analyzed by a blind investigator. The drug was injected intraperitoneally (100 μl/d PBS for the control group, 0.1 mg/kg/d Oxa for the treatment group) once a week. Tumor nodule volumes were measured every three days using the formula: *V* = π × (*d*^2^ × *D*)/2, where *d* was the minor tumor axis and *D* was the long diameter, until day 27. After euthanatized on day 28, tumors were collected and weighted.

### Quantitative real-time PCR

Total RNA was isolated using Trizol reagent (Invitrogen, California, USA) following the manufacturer’s instructions. One microgram of total RNA was reverse transcribed using Reverse Transcriptase kit (Thermo Fisher Scientific, Waltham, USA). Quantitative real-time PCR was performed with Ultra SYBR Mixture (CWBIO, China) on a CFX96 Real Time PCR Detection System (Bio-Rad, USA). The PCR primers are shown in Supplemental Table S2. The relative levels of gene expression were represented as ΔCt-Ct gene-Ct reference, and the fold change of gene expression was calculated by the 2^−ΔΔCt^ method. Each sample was practiced in triplicate. GAPDH was used as an internal control for all samples.

### RNA immunoprecipitation (RIP)

The RIP-Assay Kit (Geneseed, Guangzhou, China) was used to detect interaction of AKAP8L and SCD1 according to the manuscript’s instruction. Briefly, 5 × 10^7^ BGC-823/Oxa or MKN-45/Oxa cells were harvested and lysed in lysis buffer containing protease inhibitor cocktail and RNase inhibitor. The cell extract was incubated with 25 μl of protein A/G magnetic beads for 4 h at 4 °C (Magnetic beads were pre-incubated with anti-AKAP8L (Novus) antibody or normal rabbit IgG (CST) polyclonal antibody for 2 h at 4 °C). Next, RNA was isolated from the antibody-immobilized protein A/G beads complex. RNA enrichment was analyzed using qPCR.

### RNA degradation assay

Twenty-four hours after cell seeding, actinomycin D (ActD, Apexbio) were, respectively, used at a final concentration of 50 mM and 10 μg/mL. Cells were collected at indicated times (0, 2, 4, 6, and 8 h after actinomycin D treatment) and the expression of SCD1 and GAPDH mRNA were detected by qPCR.

### Gene-specific m6A qPCR

The MeRIP Kit (BersinBio, China) was used to investigate the expression of m6A-modified SCD1 levels according to the manuscript’s instruction. Total RNA was isolated from BGC-823/Oxa cells by Trizol extraction. Anti-m6A antibody and Protein A/G magnetic beads were added to the mixture and incubated for 4 h at 4 °C with rotation about 10 r/min. m6A RNA was purified by RNA purification kit. The m^6^A enrichment was analyzed using qPCR.

### Western blot

Total protein was isolated using RIPA buffer (Solarbio) supplemented with protease inhibitor cocktails (Sigma). The proteins were separated by SDS-polyacrylamide gel, transferred onto polyvinylidene fluoride membranes (Millipore). After blocking with 5% fat-free milk for 2 h at RT, then membranes were incubated with primary antibodies overnight at 4 °C, followed by peroxidase-conjugated secondary antibodies (1:5000 dilution) for 2 h at RT. The immune complex was visualized by an enhanced chemiluminescence kit (Millipore). The primary antibodies were used at the following dilutions: AKAP8L (1:1000, NBP2-47440; Novus), Lgr5 (1:1000, NBP1-28904; Novus), Oct4 (1:1000, NB100-2379; Novus), CD133 (1:1000, #64326; CST), CD44 (1:1000, #37259; CST), Sox2 (1:1000, #3579; CST), SCD1 (1:1000, DF13253; Affinity), IGF2BP1(1:1000, ab184305; abcam), Cleaved-PARP (1:1000, AF7023; Affinity), PARP (1:1000, #9532; CST), Cleaved-Caspase3(1:1000, #9664; CST), Caspase3(1:1000, #AF6311; Affinity), β-actin (1:2000, #4970; CST), GAPDH(1:1000, #2118; CST).

### Co-immunoprecipitation (Co-IP)

Cells were collected 24 h after transfection and lysed in lysis buffer supplemented with a protease inhibitor cocktail (Sigma), and a phosphatase inhibitor cocktail (Sigma). Supernatants were collected and incubated with the protein A/G beads (MCE) and anti-Flag magnetic beads (Sigma) overnight at 4 °C. Then, beads were washed five times with lysis buffer. Immunoprecipitates were eluted by boiling with 2 × SDS loading buffer.

### Immunohistochemical assay

Immunohistochemical assay was performed to detect the expression of AKAP8L, SCD1, and CD133 in the sections of human gastric cancer tissue arrays or mouse xenograft tissues. In brief, paraffin-embedded slices (4 μm) were deparaffinized, and then boiled in 0.01 M citrate buffer (pH 6.0) for 15 min. The endogenous peroxide activity was blocked in 3% H_2_O_2_ solution for 30 min, and the sections were incubated with 5% BSA to reduce nonspecific binding. Tissue sections were incubated with the primary antibodies (anti-AKAP8L, SCD1, and CD133, 1:100 dilution) at 4 °C overnight. After incubation with the secondary antibody for 1 h, DAB was added. The immunostaining images were captured using an Olympus microscope, mean optical density (MOD) values for each specimen were measured by Image-pro plus software.

### Immunofluorescence (IF) and TUNEL staining

The paraffin-embedded slices were dewaxed and washed with PBS, followed by permeabilization with 0.3% Triton X-100 for 30 min at RT. The coverslips were blocked with 5% BSA and incubated with anti-Ki67(1:200, ab16667; Abcam) at 4 °C overnight. or terminal deoxynucleotidyl transferase (TdT) in reaction buffer (containing a fixed concentration of digoxigenin-labeled nucleotides, TUNEL, AF594) at 37 °C for 1 h. Subsequently, the cells were incubated with fluorescence-conjugated secondary antibodies for 1 h. Then, the nuclei were stained with DAPI(Sigma). The stained cells were observed with a fluorescence microscope (Olympus, Japan).

### Bioinformatics

The correlation between the expression of AKAP8L and SCD1 was analyzed by TCGA Tumor from GEPIA Databases. BLAST was used to predict the potential binding sites between AKAP8L and SCD1 mRNA. Furthermore, the expression of AKAP8L and SCD1 and their prognosis roles in gastric cancer were confirmed by Kaplan–Meier plotter analysis.

### Statistical analysis

Sample sizes were denoted in the figure legends. All experiments were performed in triplicate. Samples included in the analyses surely met proper experimental conditions. Results were quantified as mean ± SEM. All statistical analysis were conducted with SPSS v20.0 software (Abbott Laboratories, USA) and GraphPad Prism software 6.0 (GraphPad Software, USA), and performed using the Student’s *t* test. Kaplan–Meier plotter analysis were used to estimate the prognostic relevance of AKAP8L and SCD1 in univariate analysis. Two-tailed Spearman’s correlation analysis was used to analyze the associations between AKAP8L and SCD1 mRNA expression. *P* < 0.05 was considered statistically significant.

## Results

### AKAP8L is upregulated in chemoresistant GC and predicts poor prognosis

**Fig. 1:**
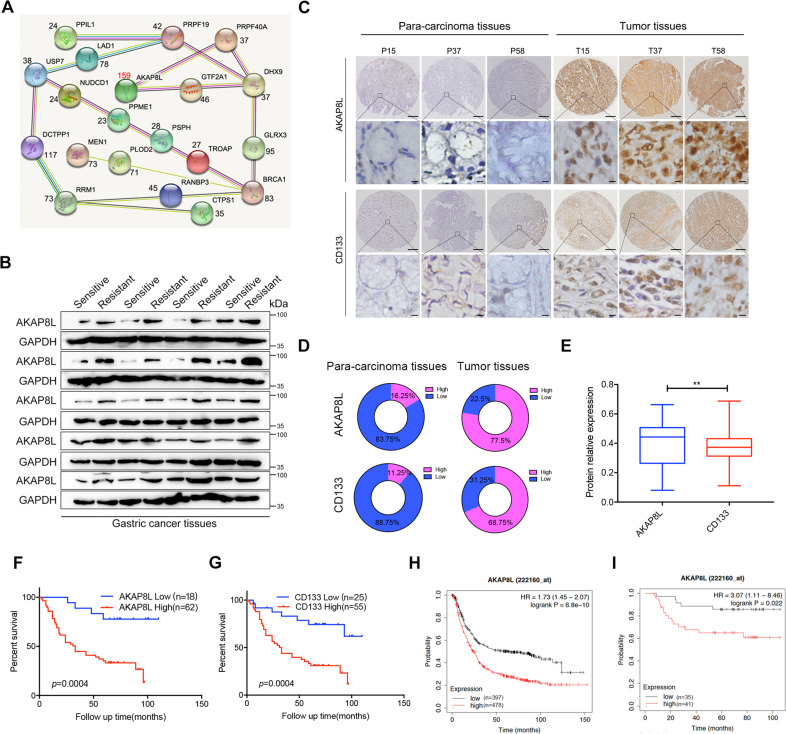
AKAP8L is upregulated in chemoresistant gastric cancer. **A** Diagram of the interactions of the top 20 upregulated genes in non-responders to chemotherapy versus responders in a cohort of 6 patients treated at Shandong Cancer Hospital and Institute. **B** Western blot analysis of AKAP8L in chemosensitive and chemoresistant gastric tumors. Representative images of AKAP8L and CD133 immunostaining in para-carcinoma tissues and gastric tumor tissues (**C**, **D**) (tissue array, *n* = 80). **E** Two-tailed Spearman’s correlation analysis of AKAP8L and CD133 expression in GC tissues. **F**, **G** Analysis of the AKAP8L and CD133 expression levels in relation to the overall survival of GC patients (tissue array, *n* = 80). **H**, **I** Analysis of the AKAP8L expression levels in relation to the overall survival (**H**) and first progression (**I**) of GC patients treated with chemotherapy regimen from the Kaplan–Meier plotter database (222160_at) (*n* = 875). Upper panel: scale bars: 500 μm. Lower panel: scale bars: 20 μm.

To determinate the GC chemoresistance-related genes, mass spectrometry was performed to investigate the differential gene expression in non-responders to chemotherapy versus responders. Top 20 upregulated genes and their interactions in chemoresistant GC specimens were shown in Fig. 1A, in which AKAP8L was the highest upregulated gene. Western blot results confirmed that AKAP8L protein level was higher in non-responders to chemotherapy, compared to responders (Fig. 1B). Expression of AKAP8L was tested in 80 pairs of GC tissues using IHC. AKAP8L protein level was much higher in GC tissues than that in para-carcinoma tissues (Fig. 1C, D). Clinical association study revealed that AKAP8L expression was significantly correlated with poor differentiation state (Pearson χ2 test, *P* = 0.0004), and the expression of CD133 (Pearson χ2 test, *P* = 0.0075) (Fig. 1C–E and Table S1). In addition, high AKAP8L and CD133 expression levels were associated with poor overall survival (OS) of GC patients (Fig. 1F, G). Kaplan–Meier survival analysis from Kaplan–Meier plotter database (222160_at) also showed that the high AKAP8L mRNA levels were significantly related to poor overall survival rates of GC patients (Fig. 1H), and the first progression of GC patients treated with chemotherapy (Fig. 1I). Collectively, AKAP8L may function as a potential prognostic marker of GC.

### AKAP8L facilitates the stemness in GC cells

**Fig. 2:**
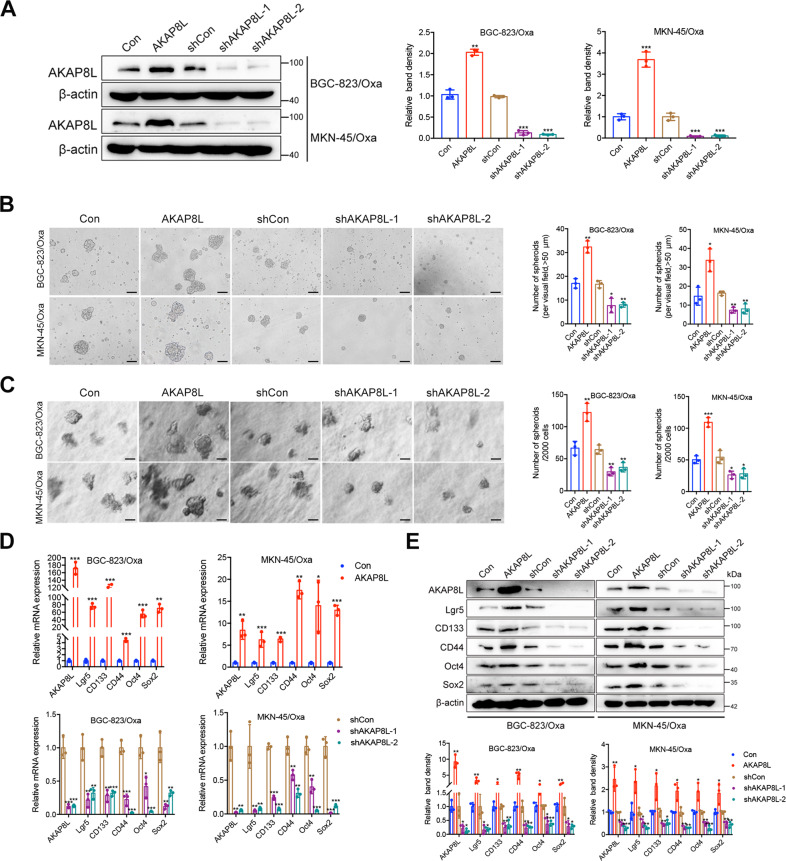
AKAP8L enhances the stemness in GC cells. **A** Western blot analysis showed ectopic expression of AKAP8L in GC cells transfected with AKAP8L (AKAP8L) or control lentivirus (Con), and AKAP8L silencing in cells treated with scrambled shRNA (shCon) or shRNA against AKAP8L (shAKAP8L-1, shAKAP8L-2). **B** Representative images and quantification analysis of spheroids in AKAP8L overexpression or the control groups, and AKAP8L knockdown or the control groups. Scale bar indicates 50 μm. **C** In soft agar colony formation assay, representative images and numbers of spheroids formed by AKAP8L-overexpressing and AKAP8L-knockdown BGC-823/Oxa and MKN-45/Oxa. Scale bar indicates 50 μm. **D**, **E** qPCR and Western blot assays showed the expression of stem cell markers induced by AKAP8L or AKAP8L shRNAs. The values indicate the mean ± standard deviation (SD) of three independent experiments. *P < 0.05, **P < 0.01,***P < 0.001.

The positive correlation of AKAP8L and CD133 indicates that AKAP8L may be involved in GC stemness. To examine the role of AKAP8L in the stemness of gastric cancer cells, BGC-823/Oxa and MKN-45/Oxa cells were transfected with AKAP8L overexpression or AKAP8L short hairpin (sh)RNA letivirus (Fig. 2A) and grew them under spheroid-formation conditions. Overexpression of AKAP8L in gastric cancer cells led to a ~52% increase in spheroid number (>50 μm) compared to that in the control cells (Fig. 2B). Two shRNAs (AKAP8L shRNA-1; AKAP8L shRNA-2) in gastric cancer cells led to a ~47% decrease in spheroid number compared to that in the control cells (Fig. 2B). These results were consistent with that of AKAL8L overexpression and AKAP8L knockdown GC cell spheroid-formation in soft agar (Fig. 2C). Ectopic AKAP8L in gastric cancer cells and spheroids elevated the expression of stem cell markers, including Lgr5, CD133, CD44, Oct4, Sox2, as determined by qPCR, Western blot analysis. Inhibition of AKAP8L obtained the opposite results (Fig. 2D, E). Thus, AKAP8L promotes the stemness of GC cells such as expression of stemness-related genes and spheroid formation.

### AKAP8L promotes the chemoresistance in GC cells

**Fig. 3:**
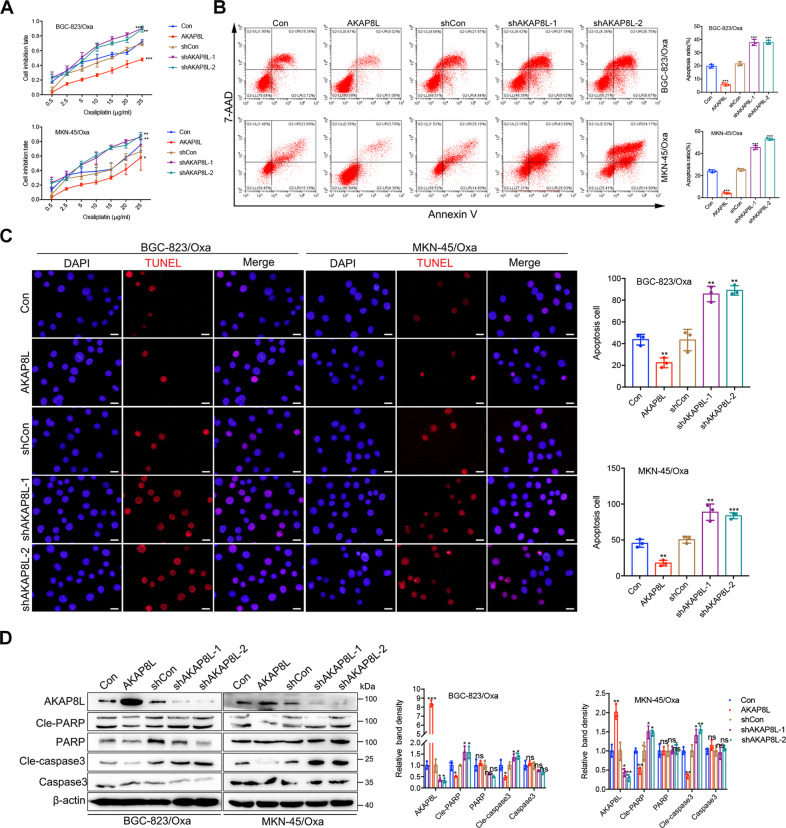
AKAP8L promotes the chemoresistance of GC cells. **A** Cell viability was measured by MTT in AKAP8L-overexpressing cells and AKAP8L silencing cells after the treatments of Oxa. **B** Representative images of flow cytometry showed the percentage of apoptotic cells in AKAP8L or AKAP8L shRNA transfected cells. **C** Representative images and quantification analysis of TUNEL assay. **D** Apoptosis-related proteins were measured in AKAP8L overexpression or AKAP8L knockdown GC cells. Scale bar indicates 50 μm. The values indicate the mean ± SD of three independent experiments.*P < 0.05, **P < 0.01,***P < 0.001.

Resistance to chemotherapy is a typical stemness-related property. After treating with a commonly used chemotherapeutic reagents, oxaliplatin, at different concentrations, cell viability of AKAP8L-overexpressing cells was significantly higher than that of controls (Fig. 3A). Flow cytometry results also revealed that the percentage of apoptotic cells was significantly lower in AKAP8L transfected cells than that in controls (Fig. 3B), which was consistent with TUNEL assay results (Fig. 3C). Downregulation of cleaved-caspase 3 and cleaved-PAPR was observed in AKAP8L overexpression GC cells (Fig. 3D). AKAP8L deficiency led to the opposite results (Fig. 3A–D). These results indicate that AKAP8L enhanced Oxa chemoresistance in vitro.

### AKAP8L promotes GC stemness and chemoresistance in vivo

**Fig. 4:**
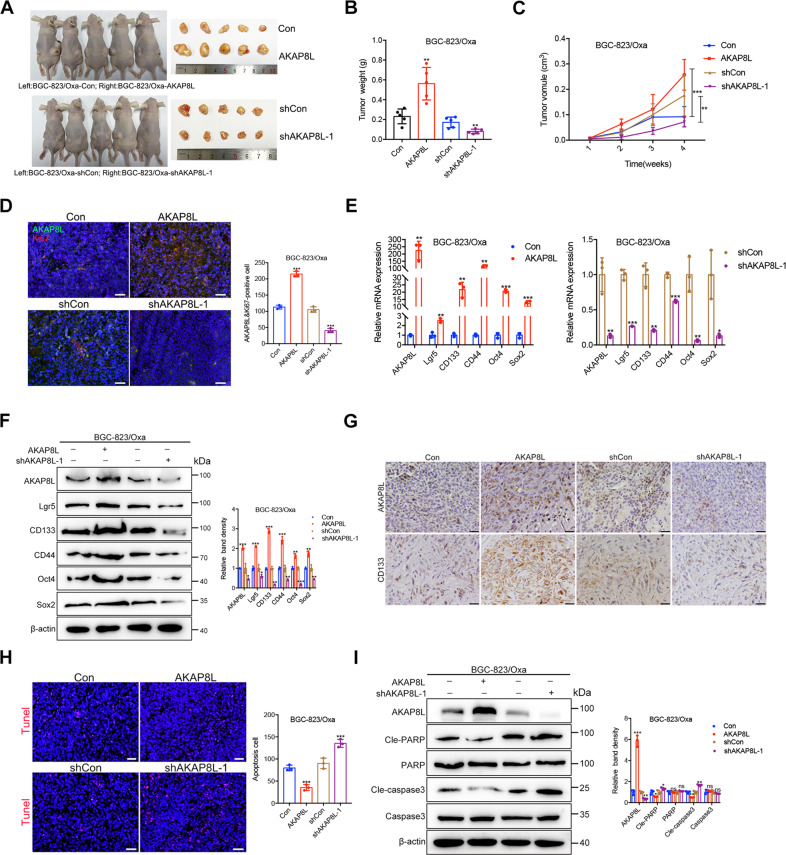
AKAP8L controls the chemoresistance in vivo. **A** 1 × 10^6^ AKAP8L-overexpressing or AKAP8L-knockdown BGC-823/Oxa were implanted in nude mice (tumor development following treatment with Oxa once a week), tumors were dissected and representative images of the tumors are shown. **B**, **C** Tumor growth curves for orthotopic models. **D** Representative images of immunofluorescence staining of AKAP8L, Ki-67 and quantification analysis of AKAP8L, Ki-67 positive cells in xenograft tumors. Scale bar indicates 50 μm. **E**, **F** qPCR and Western blot analysis of the expression of AKAP8L, Lgr5, CD133, CD44, Oct4, and Sox2 in xenograft tumors. **G** Representative immunohistochemical staining of AKAP8L and CD133 in the indicated tumor tissues. Scale bar indicates 20 μm. **H** Immunofluorescence staining of TUNEL was performed to evaluate the apoptotic cells. Scale bar indicates 50 μm. **I** Western blot analysis of cleaved-caspase 3 and cleaved-PAPR. The values indicate the mean ± SD of three independent experiments. *P < 0.05, **P < 0.01, ***P < 0.001.

We next evaluated whether forced expression of AKAP8L affected the GC chemoresistance in vivo. The effect of AKAP8L overexpression on Oxa chemotherapy was examined on BGC-823/Oxa gastric cancer xenografts in mice. Once tumors reached 100 mm^3^, mice were randomized and treated with Oxa once a week. AKAP8L accelerated tumor growth by approximately 2- to 3-fold compared with the tumors treated with Oxa (Fig. 4A–C). Tumor cell proliferation was determined by performing Ki67 immunostaining. Overexpression of AKAP8L led to significant increase of the Ki67 positive cells rate compared to AKAP8L shRNA cells. (Fig. 4D). As shown in Fig. 4E–G, ectopic AKAP8L upregulated the expression of GC stemness-related genes at mRNA level and protein levels. Inhibition of AKAP8L resulted in the downregulation of GC stemness-related genes. Overexpression of AKAP8L attenuated Oxa-induced cell apoptosis(Fig. 4H, I). Taking together, these findings suggest that AKAP8L promotes GC chemoresistance in vivo.

### AKAP8L upregulates SCD1 expression via mRNA stability in response to chemotherapeutic drug treatment

**Fig. 5:**
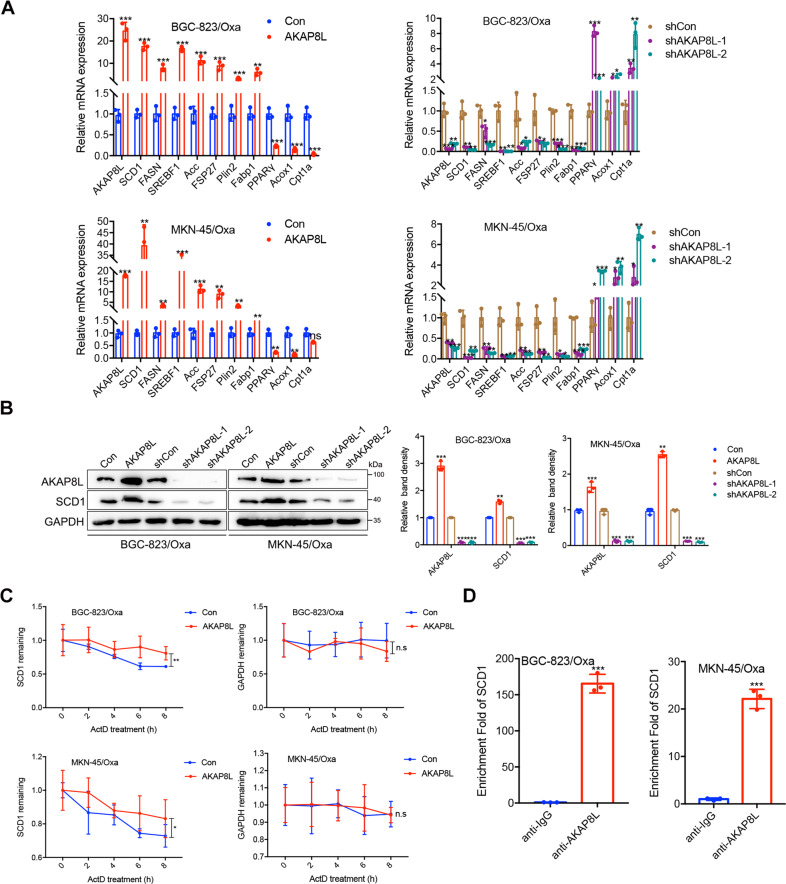
AKAP8L upregulates SCD1 expression via mRNA stability. **A** qPCR assay showed the expression of lipid metabolism-related genes. **B** Western blot analysis showed the expression of SCD1 in AKAP8L or AKAP8L shRNA transfected GC cells. **C** BGC-823/Oxa or MKN-45/Oxa cells were transiently transfected with control or AKAP8L plasmid for 48 h and then treated with ActD for 0, 2, 4, 6, 8 h. The SCD1 mRNA level was determined using qPCR. The GAPDH mRNA level was used as a negative control. **D** RIP assay using total cell lysates of BGC-823/Oxa or MKN-45/Oxa cells was used to assess the interaction between AKAP8L and SCD1 mRNA. Enrichment of SCD1 mRNA in the AKAP8L-containing immunoprecipitated particles was measured using qPCR and normalized to input. The values represent the mean ± SD of three independent experiments (*P < 0.05, **P < 0.01, ***P < 0.001 independent Student’s *t*-test).

CSCs rely highly upon lipid metabolism for maintaining their stemness properties. To exploit the effects of AKAP8L on aberrant lipid metabolism in GC cells, the genes related to lipid droplet synthesis, lipid uptake, and β-oxidation in Oxa-resistant BGC-823 and MKN-45 cells were analyzed by qPCR (Fig. 5A). Stearoyl-CoA desaturase 1(SCD1), a key enzyme that converts saturated fatty acids, was dramatically upregulated in AKAP8L transfected GC cells at mRNA level and protein level (Fig. 5A, B). AKAP8L knockdown resulted in the decrease of SCD1 expression in GC cells (Fig. 5A, B).

Since AKAP8L acts as mRNA binding protein, engaging in pre-mRNA splicing and transcription, to test whether AKAP8L affected the SCD1 mRNA stability in GC cells, AKAP8L overexpression and knockdown GC cells were treated with a RNA synthesis inhibitor, actinomycin D(ActD). Compared to the control group, AKAP8L overexpression attenuated SCD1 mRNA decay (Fig. 5C). RIP results revealed that AKAP8L band to SCD1 mRNA (Fig. 5D). Collectively, these results confirmed that AKAP8L enhanced SCD1 mRNA stability in Oxa-resistant GC cells.

### AKAP8L stabilizes SCD1 mRNA via IGF2BP1-dependent manner

**Fig. 6:**
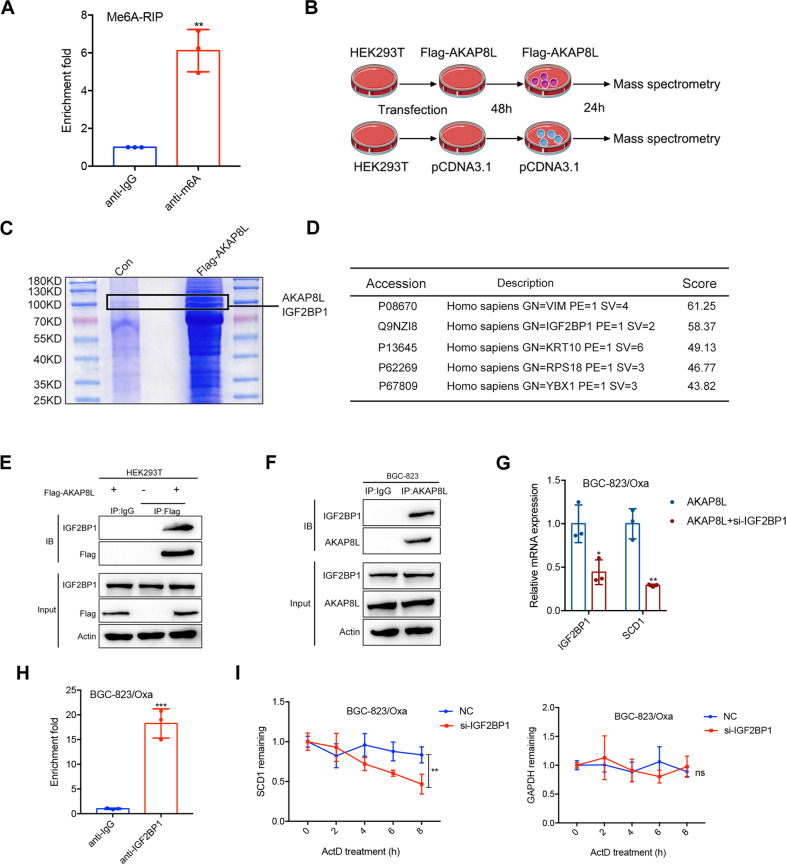
AKAP8L stabilizes of SCD1 mRNA via IGF2BP1-dependent manner. **A** m6A RIP and qPCR were used to determine the percentage of SCD1 mRNA with m6A modification in Mettl3 overexpressing cells. Affinity mass spectrometry (**B**–**D**) and Co-IP assay (**E**, **F**) were performed to detect the interaction of proteins with AKAP8L. **G** AKAP8L overexpressing cells were transiently transfected IGF2BP1 siRNA. The mRNA level of SCD1 was measured by qPCR. **H** RIP were used to determine the percentage of SCD1 mRNA with IGF2BP1 in BGC-823/Oxa cells. **I** BGC-823/Oxa cells were transiently transfected with control or siIGF2BP1 for 48 h and then treated with ActD for 0, 2, 4, 6, 8 h. The SCD1 mRNA level was determined using qPCR. The GAPDH mRNA level was used as a negative control. The values represent the mean ± SD of three independent experiments (*P < 0.05, **P < 0.01, ***P < 0.001, independent Student’s *t*-test).

m6A modification of mRNA is involved in mRNA stability. To determine whether the AKAP8L stabilized the SCD1 mRNA through the m6A-dependent modification, we performed a MeRIP-qPCR assay. The result showed that overexpression of Mettl3, an important m6A writer, in the GC cells significantly increased SCD1 mRNA m6A modification(Fig. 6A). IGF2BP1 functions as a m6A “reader”, which is associated with target mRNA stability. The results of affinity mass spectrometry and CO-IP assay showed that AKAP8L band to IGF2BP1(Fig. 6B–F). The results of qPCR showed that IGF2BP1 silence blocked the upregulation of SCD1 mRNA level induced by AKAP8L (Fig. 6G). RIP results showed that IGF2BP1 band to SCD1 mRNA (Fig. 6H). IGF2BP1 silence attenuated SCD1 mRNA stability (Fig. 6I). Collectively, AKAP8L led to an increase in SCD1 mRNA stability through IGF2BP1-dependent manner.

### SCD1 mediates the effects of AKAP8L on GC cell stemness and chemoresistance

**Fig. 7:**
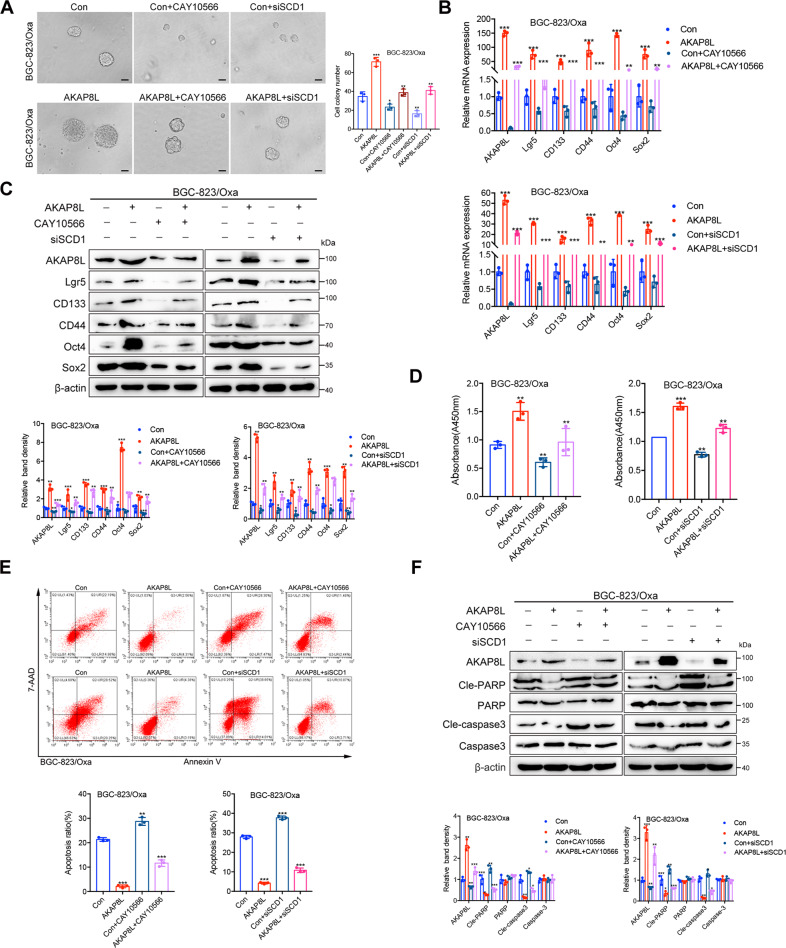
SCD1 mediates the effects of AKAP8L on GC cell stemness and chemoresistance. SCD1 siRNA was transiently transfected to AKAP8L overexpressing cells. SCD1 inhibitor (CAY10566) was added into AKAP8L overexpressing cells. **A** Representative images of spheroids in the SCD1 inhibitor, AKAP8L, AKAP8L/SCD1 inhibitor and the control groups. **B**, **C** qPCR and Western blot assays showed the stemness-related gene expression induced by AKAP8L/CAY10566 or AKAP8L/si-SCD1. **D** Cell viability was measured by MTT in four groups after the treatments of Oxa. **E** Representative images of flow cytometry showed the percentage of apoptotic cells in four groups after the treatments of Oxa. **F** Western blot analysis of cleaved-caspase 3 and cleaved-PAPR induced by AKAP8L/CAY10566 or AKAP8L/si-SCD1. Scale bar indicates 50 μm. The values indicate the mean ± SD of three independent experiments. *P < 0.05, **P < 0.01, ***P < 0.001.

To investigate whether AKAP8L facilitates GC cell stemness and chemoresistance in SCD1-dependent pathway, SCD1 siRNA or SCD1 inhibitor (CAY10566) were used in AKAP8L-overexpressing GC cells. Inhibition of SCD1 attenuated the increase of spheroid size, spheroid number, and the upregulation of the expression of stem cell markers caused by AKAP8L overexpression in GC cells (Fig. 7A–C). After the treatment of Oxa, SCD1 inhibition resulted in low cell viability and the increase of apoptotic cells in AKAP8L transfected GC cells (Fig. 7D–F). Collectively, these data showed that AKAP8L facilitated GC cell stemness and chemoresistance in SCD1-dependent pathway.

### AKAP8L were positively correlated with SCD1 in GC tissues

**Fig. 8:**
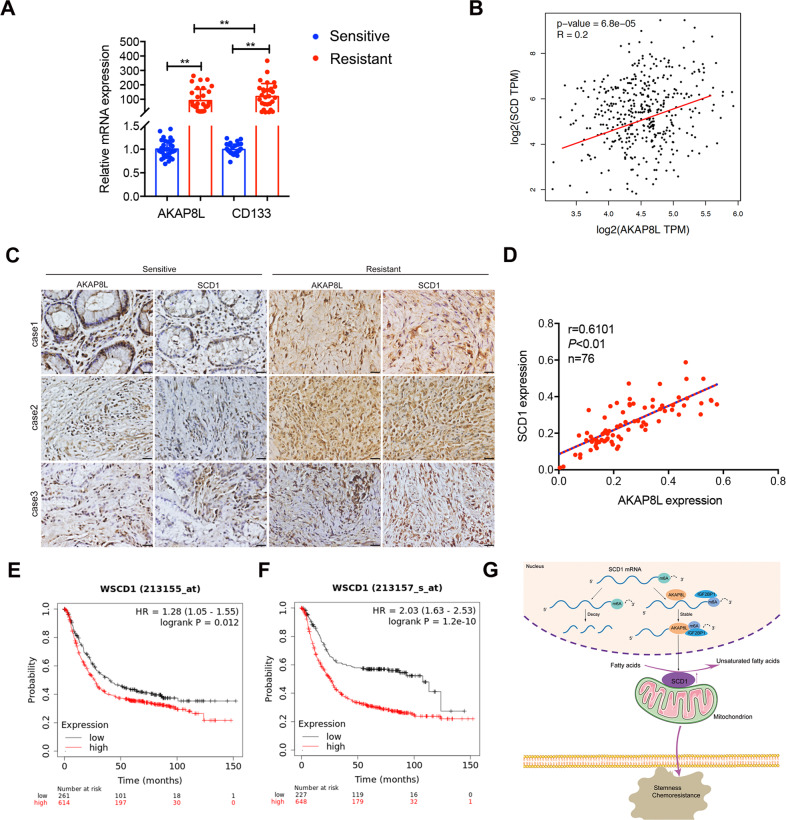
AKAP8L were positively correlated with SCD1 in GC tissues. **A** Expression of AKAP8L and SCD1 in fresh tumor tissues collected from gastric cancer patients sensitive (*n* = 31) or resistant (*n* = 45) to chemotherapy detected by qPCR. **B** Correlation between AKAP8L and SCD1 mRNA expression levels, based on GEPIA data analysis. **C** Representative images showing the expression of AKAP8L and SCD1 detected by IHC in paraffin-embedded gastric tumor tissues (*n* = 76). **D** Correlation between AKAP8L and SCD1 expression levels. Two-tailed Spearman’s correlation analysis (*n* = 76). **E**, **F** Analysis of the SCD1 expression in relation to the overall survival of GC patients from Kaplan–Meier plotter database (213155_at and 213157_s_at). **G** A schematic model showing the role of the AKAP8L in GC stemness and chemoresistance. *P < 0.05, **P < 0.01, ***P < 0.001.

Based on the above experiments, we confirmed that the stemness of GC cells was closely related to high expression of AKAP8L and SCD1, leading to enhance the chemotherapy resistance in GC cells. Therefore, the expression of AKAP8L and SCD1 was detected in chemosensitive and chemoresistant GC tissues. Fresh tumor samples were collected from 76 gastric cancer patients, including 31 patients sensitive to chemotherapy and 45 patients resistant to chemotherapy. qPCR assays showed that the mRNA expression of AKAP8L and SCD1 was significantly increased in tumors resistant to chemotherapy treatment (Fig. 8A). Further analysis from GEPIA database demonstrated that the expression of AKAP8L was positively correlated with the expression of SCD1 in GC tissues (Fig. 8B, *R* = 0.2, *P* < 0.001). Furthermore, we performed IHC on 76 paraffin-embedded gastric tumor samples, and observed the higher expression of AKAP8L and SCD1 in tumor tissues from resistant patients compared with sensitive patients (Fig. 8C). As shown in Fig. 8D, AKAP8L protein levels were positively associated with SCD1 protein levels. Consistently, the data from Kaplan–Meier plotter database (213155_at and 213157_s_at) also associated higher SCD1 expression levels with poor overall survival and first progression (Fig. 8E, F). A schematic model showed the role of the AKAP8L in GC stemness and Chemoresistance (Fig. 8G). In addition, ectopic AKAP8L upregulated the expression of SCD1 at mRNA level and protein levels in xenograft tumors (Fig. S1A–E). Inhibition of AKAP8L resulted in the downregulation of SCD1 in xenograft tumors (Fig. S1A–E). In conclusion, we confirmed that AKAP8L and SCD1 were upregulated in gastric cancer patients resistant to chemotherapy, consistent with the findings obtained from in-vitro cellular experiments.

## Discussion

Chemoresistance is the major cause of GC treatment failure [12–14]. AKAP8L was one of top upregulated genes in chemoresistant GC. Importantly, upregulated AKAP8L expression in GC cells was associated with poor differentiation state and decreased overall survival. These observations support the notion that AKAP-8L may serve as a valuable biomarker to monitor GC prognosis in humans.

AKAP8L is a member of AKAP family, which is composed of 41 members [15]. In addition to anchor PKA in nucleus, the function of AKAP8L remains largely unknown. AKAP8L and AKAP8 share 61% protein sequence identity [16]. Both proteins are engaged in pre-mRNA splicing, the initiation of replication, and the regulation of histone deacetylation and gene expression. AKAP8 plays an important role in tumorigenesis by supporting cancer cell growth [17, 18]. The N-terminal region of AKAP8L interacts with mTORC1 in the cytoplasm and mediates mTORC1 effects on protein synthesis, cell growth and cell proliferation [11]. In this study, our data showed that AKAP8L promoted GC chemoresistance for the first time.

Cancer stem cells (CSCs) possess an unlimited capacity for self-renewal, and are responsible for therapeutic resistance, disease recurrence, and tumor metastasis [19–23]. The conventional anticancer agents kill most tumor cells and fail to eliminate CSCs, and surviving CSCs can re-establish a tumor [24–26]. Considering the close relationships between CSCs and drug resistance, we therefore hypothesized that AKAP8L might participate in the GC stemness to confer chemoresistance. In the present study, AKAP8L overexpression promoted sphere-formation ability, and increased the expression of Lgr5, CD133, CD44, Oct4, and Sox2, which are essential stemness-related genes that maintain CSC-like properties in GC. Conversely, AKAP8L knockdown repressed GC stemness. We further confirmed that the regulation of GC stemness by AKAP8L is one of the major mechanisms underlying chemotherapy resistance. These results indicate that AKAP8L acts as an important promotor of GC stemness and chemoresistance.

Emerging evidences have identified that CSCs are characterized by rewiring lipid metabolism [27–29]. Dysregulated lipid metabolism is crucial for maintaining CSC stemness properties and fulfilling their energy demands [30, 31]. CSC fate decision is reliant on the activity of enzymes involved in lipid metabolism, such as SCD1 [32]. SCD1 is a key enzyme to convert saturated fatty acids into monounsaturated fatty acids (MUFAs), which is involved in the tumorigenesis of multiple cancers, including gastric cancer [33]. High expression of SCD1 might predict poor prognosis in gastric cancer patients [34]. It has been reported that SCD1 modulates GC stem-like properties and promotes tumor metastasis via Hippo/YAP pathway [35]. The regulation of SCD1 expression has not been clearly understood. In the present study, forced AKAP8L expression in gastric cancer cells dramatically enhanced SCD1 mRNA level by maintenance SCD1 mRNA stability. SCD1 mediated the effects of AKAP8L on the GC stemness and chemoresistance. Thus, our study represented the first report that AKAP8L augmented the GC stemness and chemoresistance via SCD1-dependant pathway.

N6-methyladenosine (m6A) in mRNA is one of the most prominent RNA modifications, which is associated with the regulation of post-transcriptional expression of gene [36, 37]. m6A modification is generated by the methyltransferases (m6A “writer”), the demethylases (m6A “erasers”) and effector proteins (m6A “readers”) [38, 39]. Our MeRIP result showed that Mettl3 augmented SCD1 mRNA m6A modification, which indicates SCD1 mRNA stability could be regulated via m6A modification dependent manner. IGF2BP1 is known as a m6A reader, which has been reported to stabilize target mRNA [40, 41]. We observed that AKAP8L interacted with IGF2BP1, and IGF2BP1 band to SCD1 mRNA. Importantly, SCD1 mRNA stability was significantly attenuated when IGF2BP1 was silenced. Thus, our study provided the evidence that AKAP8L promoted SCD1 mRNA stability through IGF2BP1-dependent manner.

In the clinical setting, our data demonstrated that AKAP8L and SCD1 were upregulated in gastric cancer patients resistant to chemotherapy. In consistent with the results of GEPIA database analysis, our results confirmed that AKAP8L expression was positively associated with SCD1 expression in GC tissues. Further research on the inhibition of AKAP8L could provide opportunities for AKAP8L-targeted therapies in chemoresistant gastric cancer.

In summary, our data showed the role of AKAP8L in Oxa-resistant gastric cancer cells. Further experiments revealed the regulatory mechanism of the AKAP8L/SCD1 pathway on GC stemness and chemoresistance. Therefore, our study identifies AKAP8L as a prognostic marker and a therapeutic target for overcoming chemoresistance in gastric cancer.

## Supplementary information

Original western blot of Figures

aj-checklist

Supplementary table

## Data Availability

The datasets generated or analyzed during the current study are included within the article and available from the corresponding authors on reasonable request.
