# Supplementary material for: AKAP8L enhances the stemness and chemoresistance of gastric cancer cells by stabilizing SCD1 mRNA
Source: Cell Death Dis. 2022 Dec 15;13(12):1041. doi: 10.1038/s41419-022-05502-4 (PMC9755141; doi:10.1038/s41419-022-05502-4)

Original western blot of Figure 1


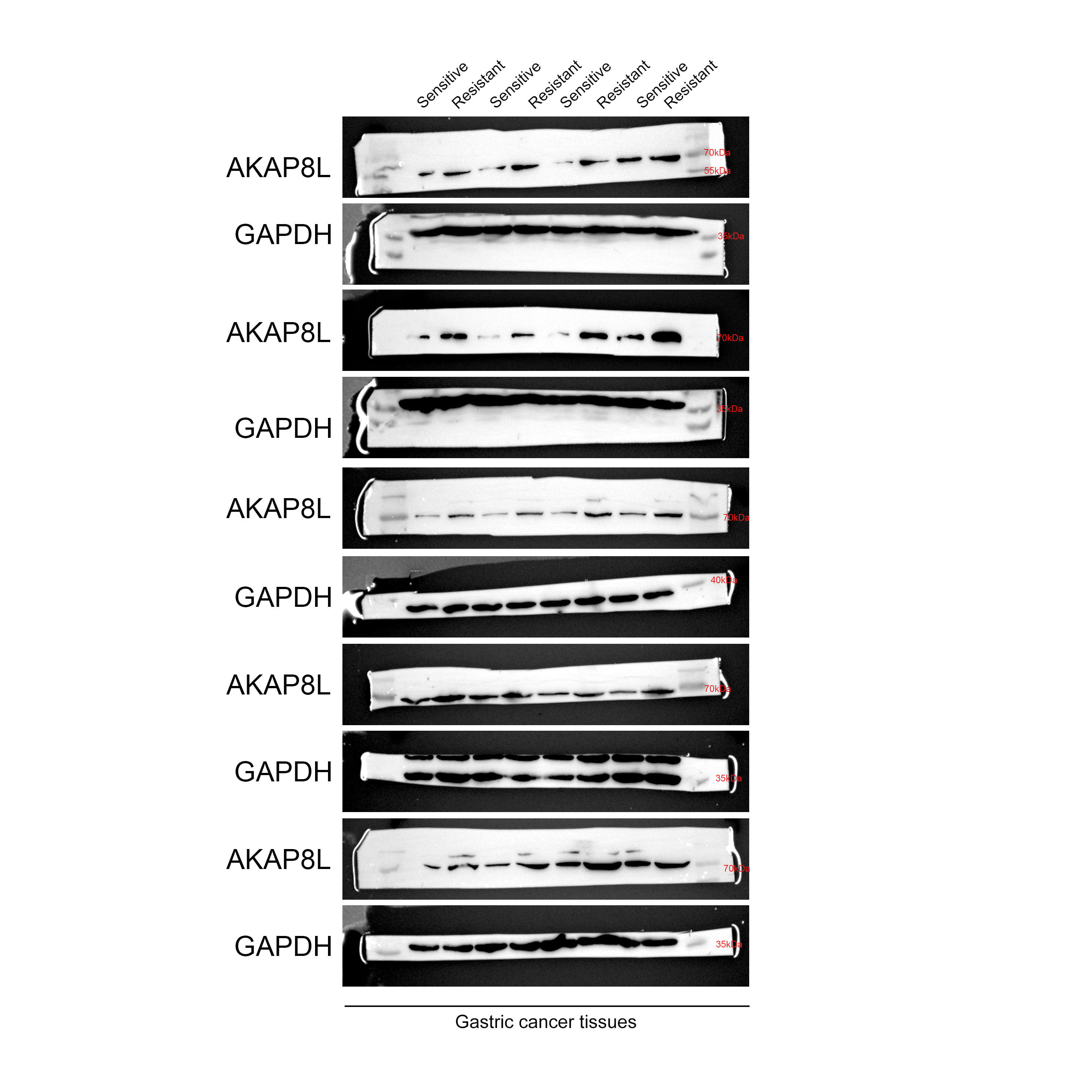


Original western blot of Figure 2


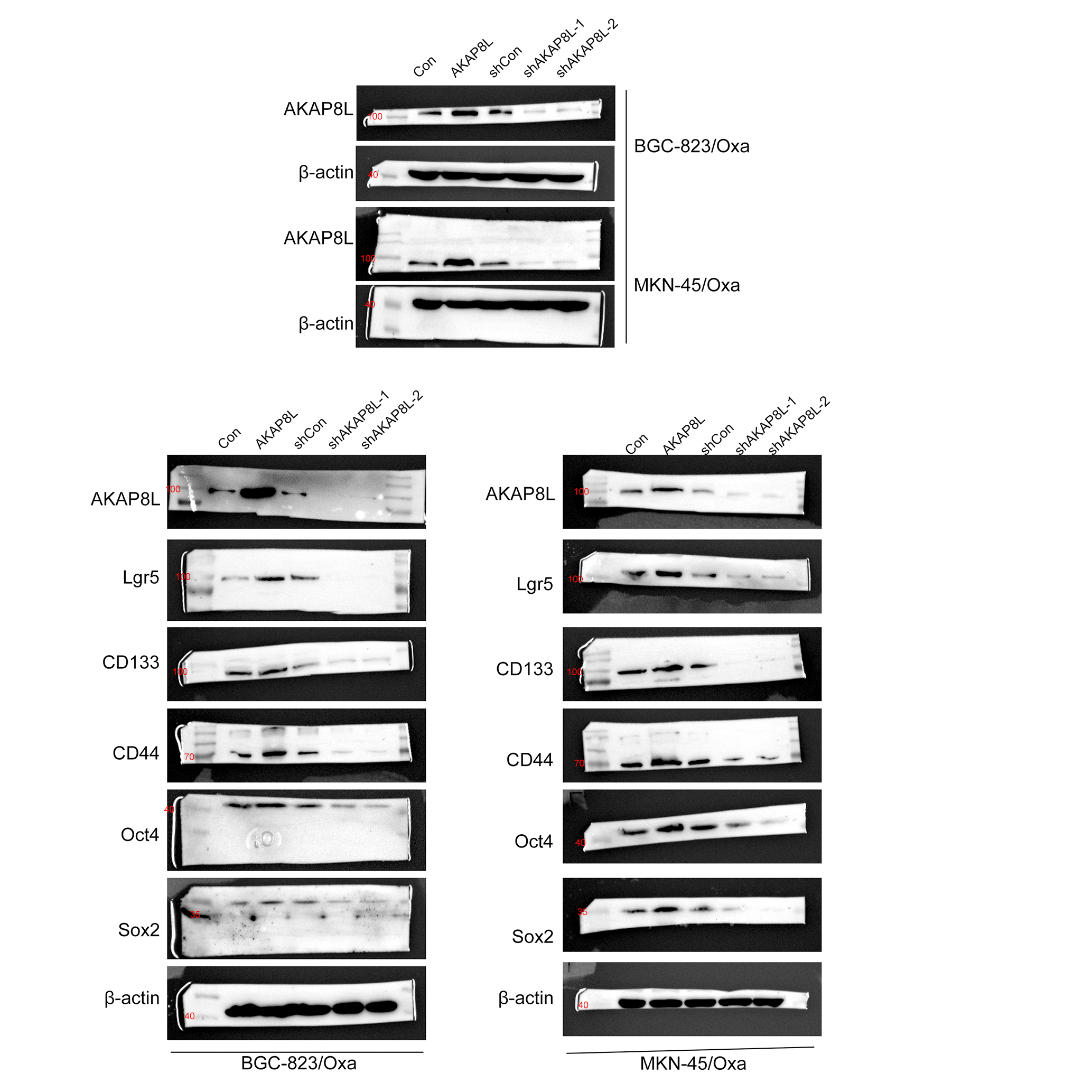


Original western blot of Figure 3


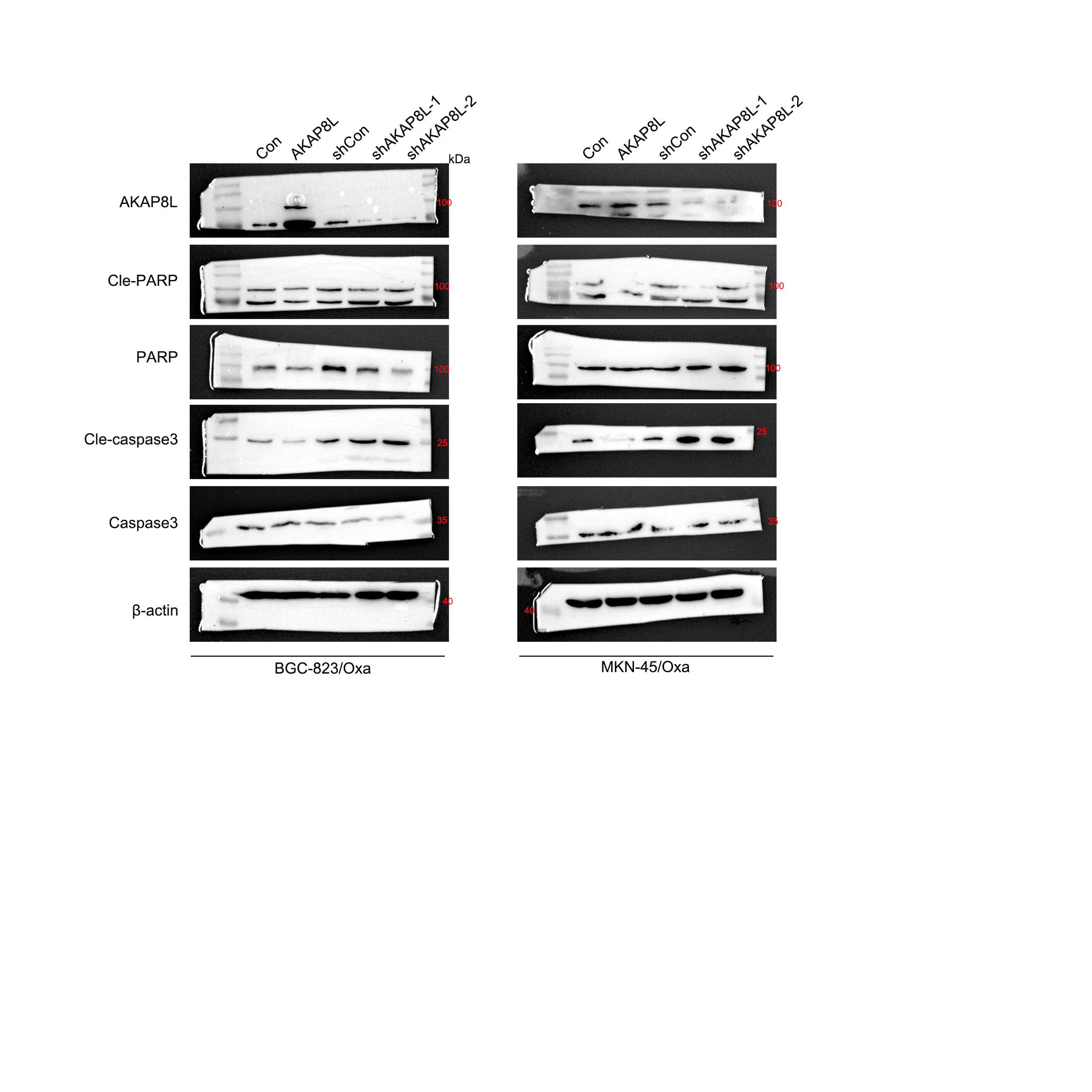


Original western blot of Figure 4


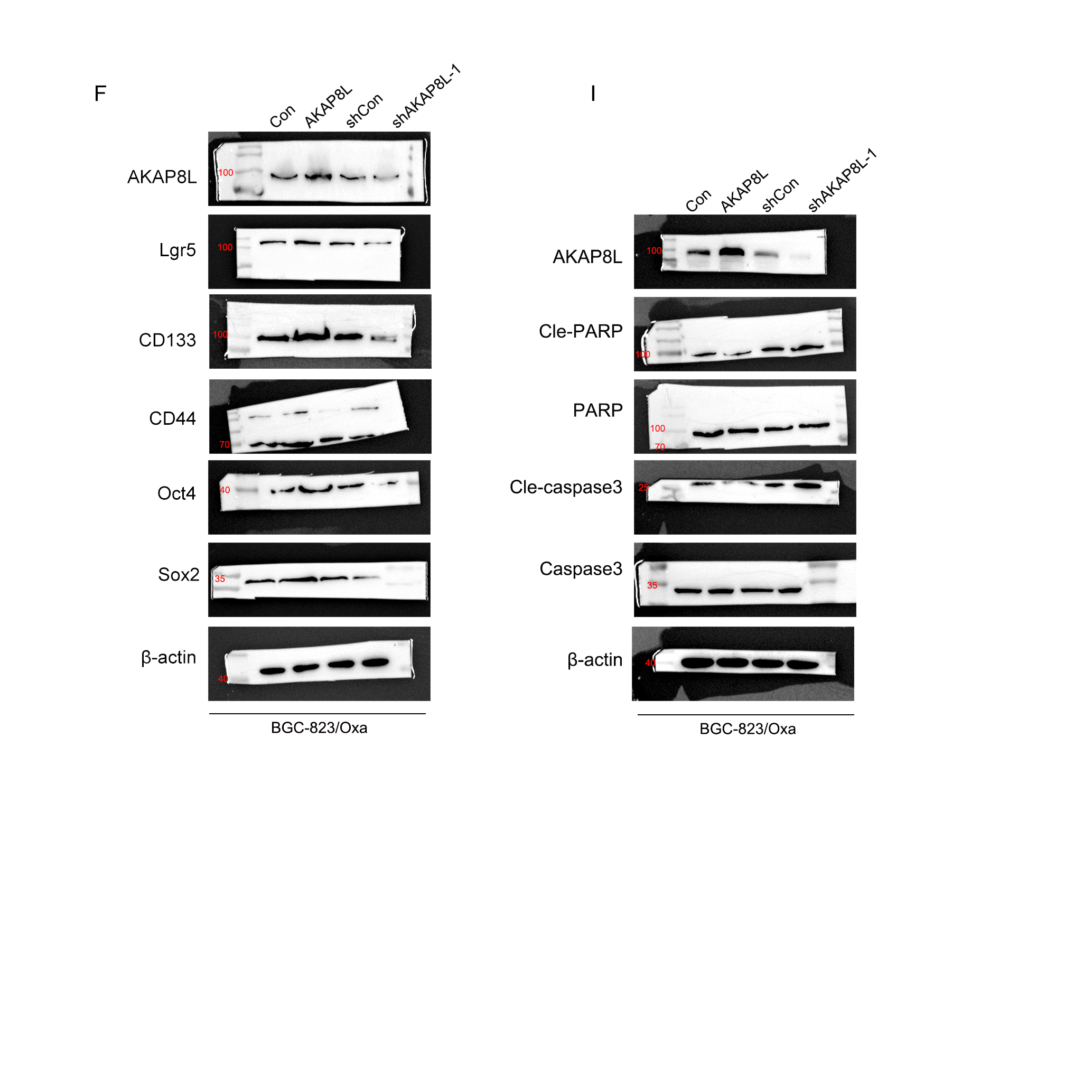


Original western blot of Figure 5


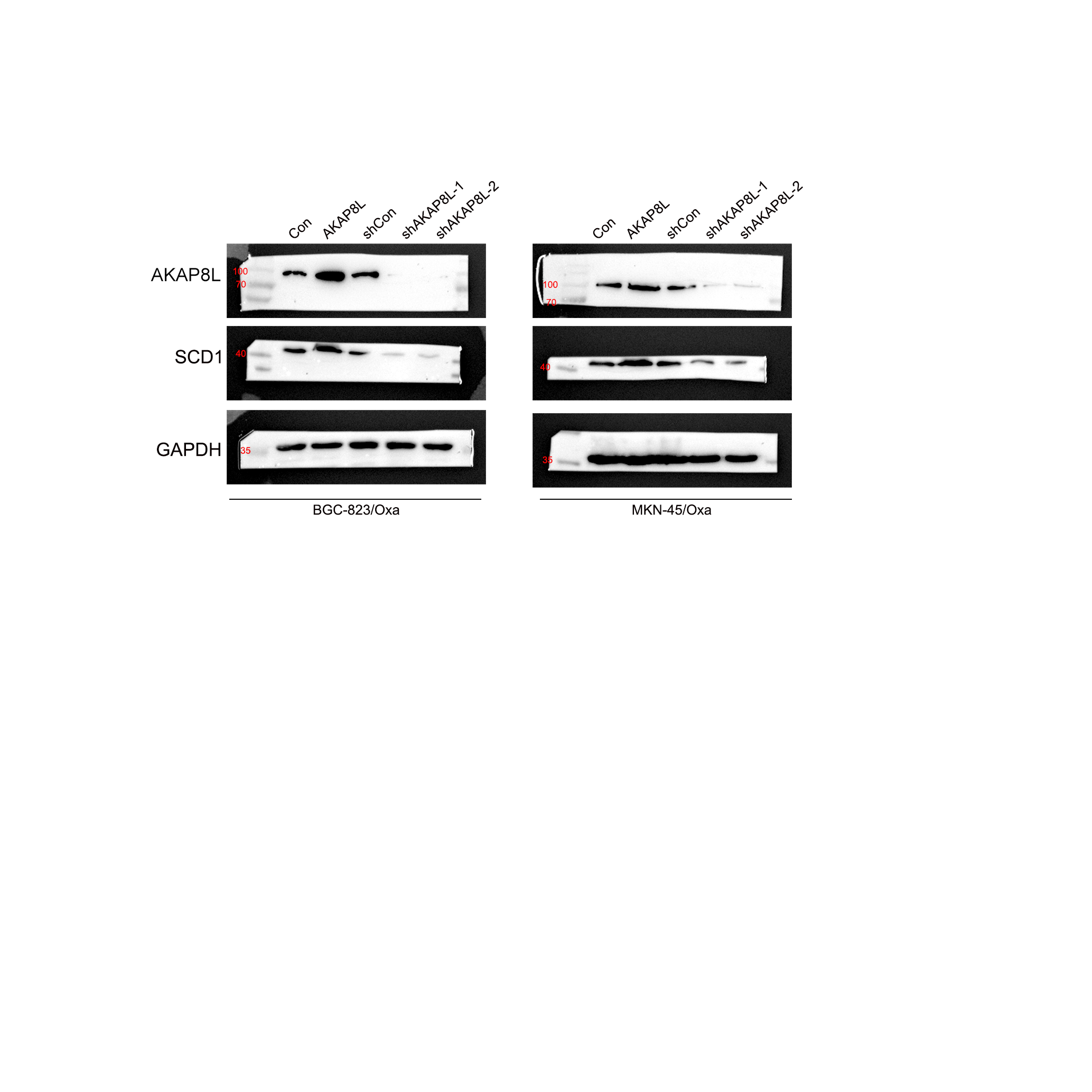


Original western blot of Figure 6


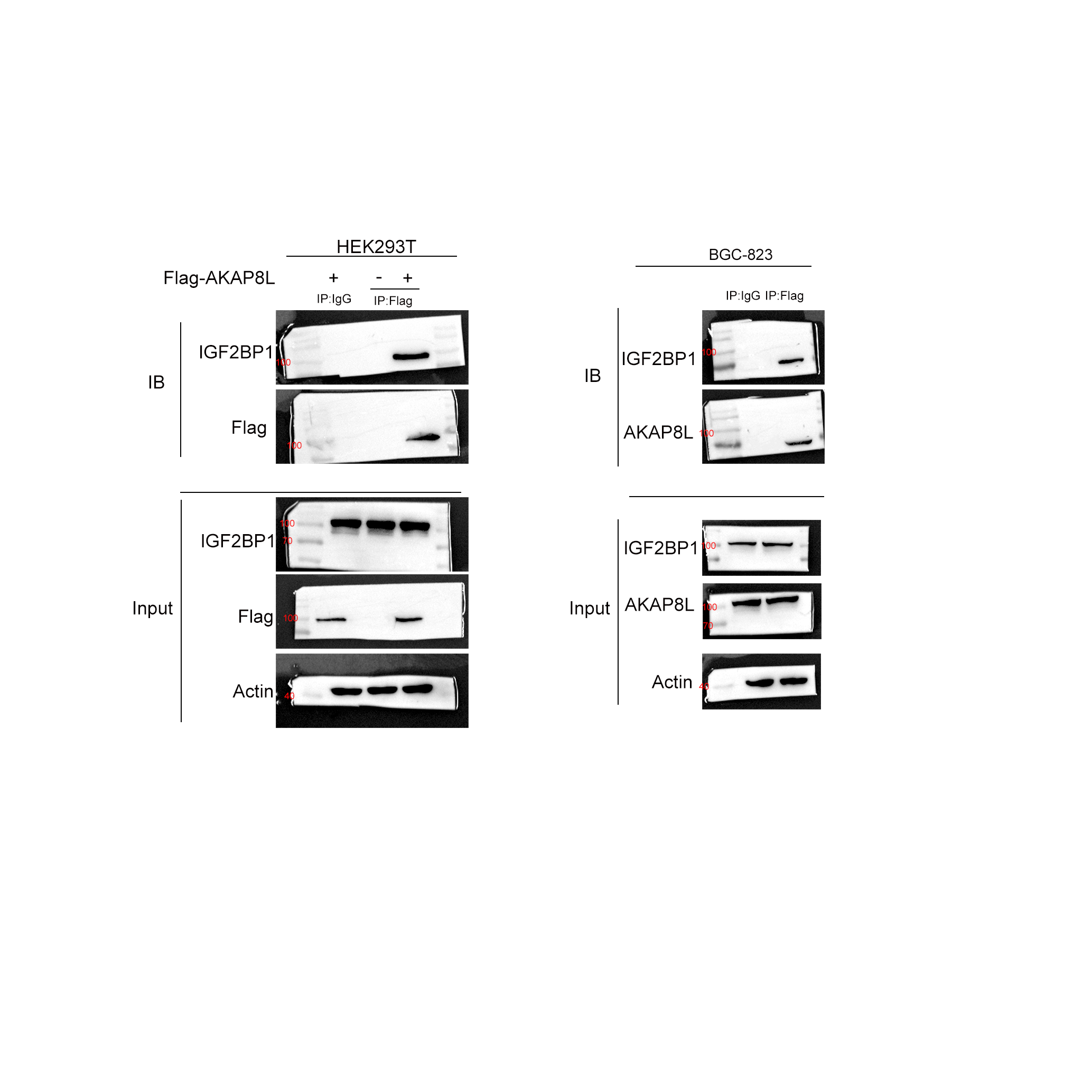


Original western blot of Figure 7


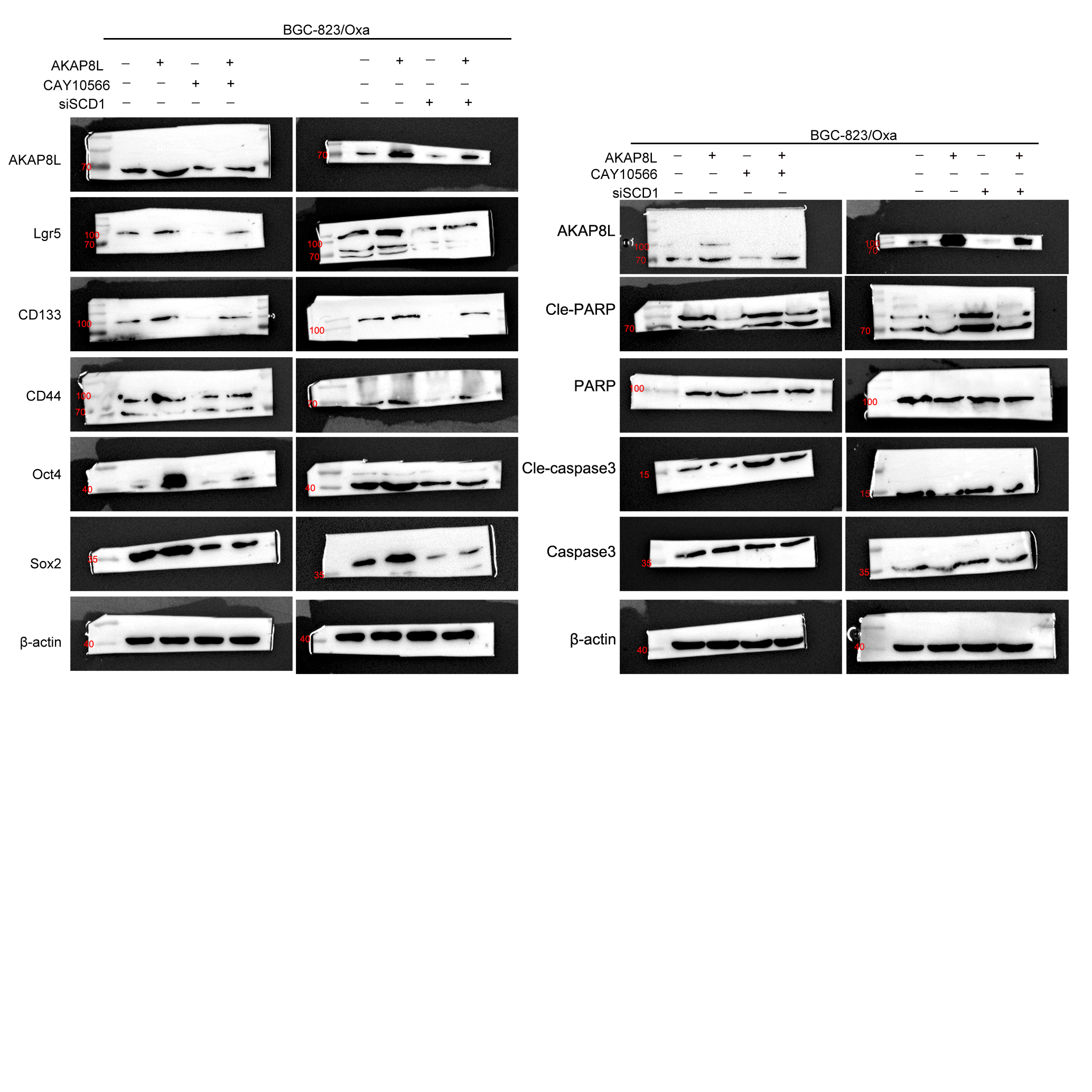

Supplement: Supplementary file 1 — Original western blot of Figures [file 41419_2022_5502_MOESM1_ESM.docx]
