# Supplementary material for: AKAP8L enhances the stemness and chemoresistance of gastric cancer cells by stabilizing SCD1 mRNA
Source: Cell Death Dis. 2022 Dec 15;13(12):1041. doi: 10.1038/s41419-022-05502-4 (PMC9755141; doi:10.1038/s41419-022-05502-4)
Supplement: Supplementary file 3 — Supplementary table [file 41419_2022_5502_MOESM3_ESM.docx]

**Supplementary Table S1: The correlation of AKAP8L expression and clinical characteristics**

**
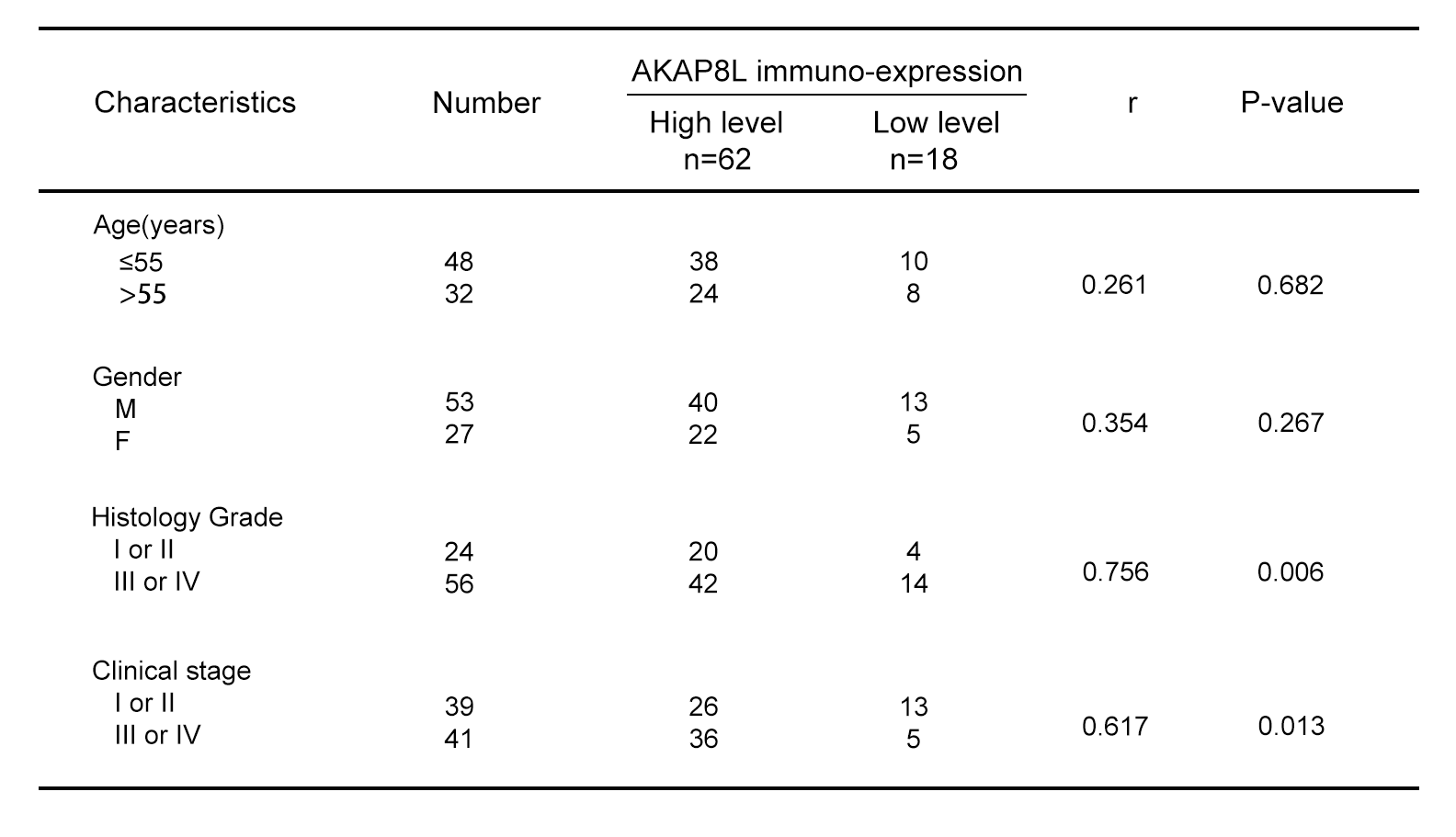
**

**Supplementary Table S2: Primer sequence for qPCR**

| **Gene** | **Forward primer** | **Reverse primer** |
| --- | --- | --- |
| GAPDH | GAAAGCCTGCCGGTGACTAA | AGGAAAAGCATCACCCGGAG |
| AKAP8L | ACCACCAACTATGGGTATGACT | GCCCTCATAGGCCATTTCCA |
| CD44 | GATCACCGACAGCACAGACA | GCCTCTTGGTTGCTGTCTCA |
| CD133 | GAGCTAAGGGAAGGGCGG | TTCTGTCTGAGGCTGGCTTG |
| Lgr5 | TTGCGAAGCCTTCAATCCCT | GGAATGCAGGCCACTGAAAC |
| Sox2 | AGGATAAGTACACGCTGCCC | TAACTGTCCATGCGCTGGTT |
| Oct4 | AAATAGCACTTCTGTCATGCTG | TATCGAGCACCTTCTATAAGCC |
| SCD1 | AAGTGCCTCACCTCGAAAGG | TGCCCTAGGCTGTAGGGAAT |
| FASN | CCTGGCTGCCTACTACATCG | CACATTTCAAAGGCCACGCA |
| SREBF1 | TAGGAAGGGCCGTACGAGG | CTTCGATGTCGGTCAGCAGC |
| Acc | CTCTTGGCCTTTTCCCGGTC | ATCAAAAGTCAGGCAAGCGG |
| FSP27 | GACTAGGAACCCTGAAGCCA | TGAACACTGTATCCCCTGCC |
| Plin2 | AGTGTCTGACAGCCTCCTCA | AAGGGACCTACCAGCCAGT |
| Fabp1 | CACCATGAGTTTCTCCGGCA | CCCATTCTGCACGATTTCCG |
| PPARγ | CCAGAAGCCTGCATTTCTGC | TGGCATCTCTGTGTCAACCA |
| Cpt1a | GTTTCTGGCTCGTTGGATGC | CCACAGCTTGGTGAGCTTCT |
| Mcad | TTCGGGGAGTATGTCAAGGC | CTGCAGCATCGCCCGAAC |


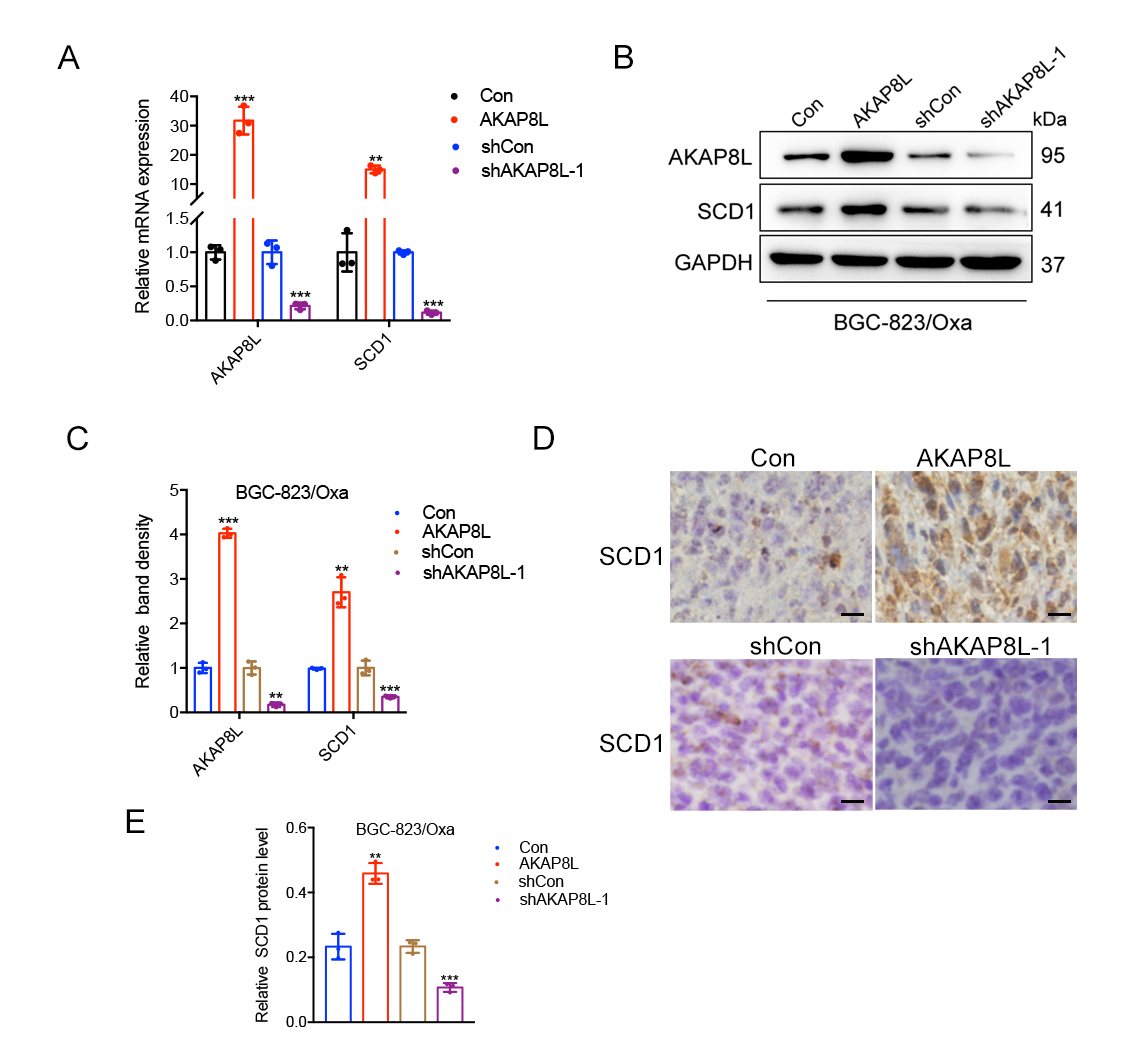


**Supplementary Figure S1. Ectopic AKAP8L upregulated the expression of SCD1 at mRNA level and protein levels in xenograft tumors.**

(A-C) qPCR and Western blot analysis of the expression of AKAP8L, SCD1 in xenograft tumors. (D,E) Representative immunohistochemical staining of SCD1 in the indicated tumor tissues. Scale bar indicates 20 μm.
